# Efficacy and Related Factors of Ultrasound-guided Lauromacrogol Injection in Treating Symptomatic Hepatic Cysts with a Diameter of <10 cm: A Retrospective Study

**DOI:** 10.2174/0115734056356792250610102812

**Published:** 2025-06-23

**Authors:** Qingyin Fu, Bin Hu, Jixian Lin, Qiping Liu, Tonghui Yang, Qiong Chen

**Affiliations:** 1 Department of Ultrasound, Minhang Hospital, Fudan University, Shanghai, China; 2 Department of Neurology, Minhang Hospital, Fudan University, Shanghai, China; 3 Department of Radiology, Dahua Hospital, Xuhui District, Shanghai, China

**Keywords:** Hepatic cyst, Lauromacrogol, Ultrasound-guided puncture, Likert scale, Sclerotherapy, Logistic regression analysis

## Abstract

**Aims:**

This study aimed to retrospectively analyze the curative effect and influencing factors of lauromacrogol in the treatment of symptomatic hepatic cysts of <10 cm.

**Methods:**

In this study, a total of 51 patients with symptomatic hepatic cysts (maximum diameter ranging from 5 cm to 10 cm) were included. Polycystic Liver Disease Questionnaire (PLD-Q) was used to evaluate the symptoms of patients prior to treatment. The patients were followed up at 1, 3, 6, and 12 months after treatment. At the 12-month follow-up, patients were asked to fill out the PLD-Q to assess their symptoms. The improvement rate of patients' symptoms was evaluated using a 5-point Likert scale (worse, 1; slight difference, 2; roughly the same, 3; good, and 4; better, 5. Volume reduction rate (VRR) was calculated by measuring the volume of the cyst cavity via ultrasound. Treatment success at the 12-month follow-up was determined using two criteria: symptom improvement and changes in cyst volume. Symptom improvement was assessed using a Likert Scale, with a score greater than 3 points indicating significant improvement. Additionally, a volume reduction rate (VRR) of 50% or more in cyst size (VRR ≥ 50%) was considered an effective treatment outcome. The relationship between the clinical factors and the ultrasonographic manifestations of hepatic cysts, including the initial maximum diameter of the cyst (measured using ultrasound before operation), the initial volume of the cyst, and the formation of septa after sclerosis of the cyst, was analyzed.

**Results:**

All patients completed at least 12 months of follow-up. After a 12-month follow-up, the effective and ineffective rates were 96.1% (49/51) and 3.9% (2/51), respectively. The logistic regression univariate analysis showed significant differences in the initial cyst volume (p = 0.001), the initial maximum diameter of the cyst (p = 0.005), and the interval formation after cyst sclerosis (p = <0.001) between VRR ≥ 50% and VRR < 50%. Logistic regression analysis demonstrated that septa formation after cyst sclerosis was an independent factor related to treatment failure, with an odds ratio of 3.246 (95% confidence interval, 0.784–4.148).

**Conclusion:**

Lauromacrogol is an effective method for hepatic cyst treatment. Septa formation after cyst sclerosis is an independent factor related to ineffective treatment.

## 1 INTRODUCTION

A hepatic cyst is a benign liver lesion that is commonly encountered in clinical practice and is generally attributed to congenital causes [1, 2]. Hepatic cysts are generally asymptomatic, and most of them are isolated cystic lesions found incidentally. When the size of a hepatic cyst increases, it induces discomfort in the right upper abdomen, pain in the liver region, abdominal distension, dull abdominal pain, and other clinical symptoms, including a large palpable abdominal mass. In severe cases, cyst bleeding and infection may occur [3]. In the past, surgery was used to remove symptomatic cysts effectively; however, its disadvantages include liver trauma, massive intraoperative blood loss, and high treatment costs [4]. The application of ultrasound-guided puncture sclerotherapy has dramatically changed this situation. Through ultrasound-guided percutaneous puncture and catheter drainage, the cyst’s contents are extracted, and then a sclerosing agent is injected, which promotes sclerosis and cyst absorption. This method is relatively simple, poses minimal trauma, enables rapid postoperative recovery of patients, and has been widely used in hepatic cyst treatment [5].

Lauromacrogol is a commonly used intravascular sclerosing agent and one of the main sclerosing agents for hepatic cyst treatment. Lauromacrogol can act on vascular endothelial cells and epithelial cells on the cyst wall, inducing aseptic inflammation and fibrosis [6, 7], and has attained optimal results for treating hepatic cysts.

In most studies, the only indicator for evaluating the efficacy of sclerotherapy is cyst volume reduction [8]. Only a few studies have used symptom relief to evaluate the efficacy of sclerotherapy [9], but none have used it as the primary research indicator. A validated polycystic liver disease questionnaire (PLD-Q) has been developed, which can be used to assess the frequency of and discomfort related to disease-specific symptoms in PLD [10]. Some studies have suggested that the symptoms of simple cysts are consistent with those of PLD [11]; thus, PLD-Q is used to evaluate the clinical efficacy of lauromacrogol sclerotherapy in patients with hepatic cysts. In this study, cyst volume reduction was used as the evaluation index of the efficacy of sclerotherapy, and the symptom improvement rate of patients was evaluated by Likert Scale after the operation.

The primary aim of this study was to evaluate the efficacy of ultrasound-guided lauromacrogol sclerotherapy for symptomatic hepatic cysts and assess factors associated with its efficacy.

## 2 MATERIALS AND METHODS

### 2.1 Study Participants and Study Design

**Fig. (1):**
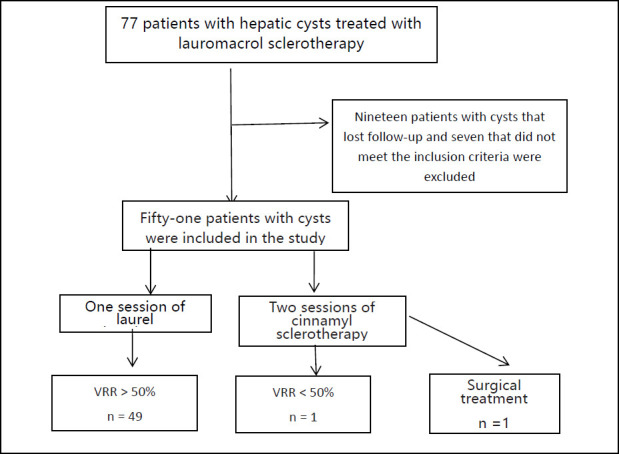
Results of ultrasound-guided lauromacrogol ablation in 77 patients with hepatic cysts. **Abbreviation:** VRR = volume reduction rate.

Data collection was conducted by three trained research assistants who were blinded to the study outcomes. This blinded approach was specifically implemented to minimize potential biases during the data extraction process, thereby enhancing the objectivity and reliability of the collected data. The subsequent data analysis was performed by senior physicians from the Department of Ultrasound Medicine. These physicians, with their extensive experience in ultrasound imaging and diagnostics, interpreted the data accurately and ensured the clinical relevance of the findings. A total of 77 patients with symptomatic hepatic cysts underwent ultrasound-guided lauromacrogol sclerotherapy at our hospital from January 2018 to August 2022. A total of 77 patients received sclerotherapy (Fig. **1**). Among them, 19 were lost to follow-up, and 7 did not satisfy the inclusion criteria and were excluded. Finally, in this study, 51 patients with cysts were included. There were 29 males and 22 females, with a male-to-female ratio of approximately 1.3:1. Before sclerotherapy, all patients were diagnosed with hepatic cysts by a multidisciplinary team comprising gastroenterologists and radiologists. Patients with symptomatic, nonparasitic, infectious, or malignant hepatic cysts were excluded. The inclusion criteria were as follows: 1) ultrasound showing complete and smooth cystic lesion capsule, without solid processes or septa in the cyst, and the cyst fluid permeable to sound; 2) those with a maximum cyst diameter of 5 cm to 10 cm; 3) those with cystic lesions presenting with symptoms, such as abdominal distension, abdominal pain, and discomfort in the liver region); and 4) those with normal liver function. The exclusion criteria were as follows: Patients 1) showing other suspected malignant lesions, 2) uncooperative with the puncture procedure, 3) with abnormal coagulation function, 4) pregnant women, and 5) those lost to follow-up within 12 months. To diagnose and measure hepatic cysts, ultrasound was used. The hepatic cyst volume was calculated using the following formula: V = πabc / 6, (V= the volume; a= front and back diameter [cm]; b= left and right diameter [cm]; c= upper and lower diameter [cm]). This study was reviewed and approved by the ethics committee of the Fudan University Minhang Hospital. Patient data is anonymised, and all patient data is protected by encryption and secure storage. This study adhered to the SAGER guidelines to ensure gender equity and transparency in our research.

### 2.2 Preoperative Preparation for Lauromacrogol Sclerotherapy

All patients were hospitalized. Then, complete routine blood tests, coagulation, liver and kidney function tests, electrocardiogram, and other routine examination procedures were performed before surgery. Patients were asked about their drinking history and drug allergy in detail. Before cyst sclerotherapy, treatment risks and possible complications after treatment were explained to the patients and their families in detail. Consent was obtained from patients and their families. Patients completed the PLD-Q to assess their symptoms before surgery. The PLD-Q questionnaire, which can be accessed online (https://www.uzleuven.be/nl/polca), is comprised of 16 items, with each item scored on a scale of 0 to 5. The questionnaire includes four distinct subscales: severity of perceived illness (SPI) with a score range of 0 to 35 and 7 items, gastroesophageal reflux disease-related complaints with a score range of 0 to 20 and 4 items, impact on food intake with a score range of 0 to 10 and 2 items, and perception of enlarged liver volume with a score range of 0 to 15 and 3 items.

### 2.3 Lauromacrogol Sclerotherapy Process

Two physicians (Dr. Fu and Dr. Yang) with at least 5 years of experience conducted all lauromacrogol sclerotherapy procedures. The patients were placed in the supine or left lateral position, and the Italian MyLab 90 color Doppler ultrasound diagnostic instrument was utilized for the preoperative abdominal examination of patients with hepatic cysts. The preoperative condition of hepatic cysts was recorded in detail, the cyst diameter and the number of intrahepatic cysts were measured in different sections, and the patients were asked whether they had a history of chronic hepatitis. The cyst location, the anatomical relationship between the cyst and the surrounding organs, and important liver structures were ascertained. The puncture path avoided the gallbladder, large intrahepatic vessels, gastrointestinal tract, and diaphragm, and the appropriate puncture point and puncture route were formulated. The skin was routinely disinfected with iodophor on the puncture site, a sterile towel was employed, and the probe was wrapped with a disposable protective sleeve. During the puncture, the patient was asked to hold his breath. Under ultrasound guidance, an 8-Fr pigtail catheter (containing the trocar core) was immediately inserted into the cyst. The needle core was removed, and the drainage tube was pushed into the cyst concurrently to ensure that the drainage tube was inserted into a 10-15cm depth (to ensure that the side holes had completely entered the cyst). The trocar core was removed, the end clamp of the drain was locked, the tee was connected, and the capsule’s contents were aspirated using a 50-mL syringe as much as possible. A bacteriological and cytological examination of the aspirated specimen was conducted. The cyst fluid color was observed. Usually, the cyst fluid color of simple hepatic cysts is pale yellow or colorless. If the color is grass green, the cyst fluid contains bile, and the cyst may have communicated with the bile duct. Contrast-enhanced ultrasound can distinguish whether a cyst is connected with the bile duct. During the operation, the cystic cavity and surrounding bile duct were visualized to determine whether the cavity was connected with the intrahepatic bile duct. If they were found to be connected, then the operation was terminated immediately. If the contrast agent was only confined to the cyst cavity and did not communicate with the surrounding bile ducts, the operation was continued, the cyst fluid was drained, and the cyst volume was recorded. After draining the cyst fluid, the cyst cavity was then repeatedly rinsed with 0.9% normal saline solution. Lauromacrogol (China Shanxi Tianyu Pharmaceutical Co., Ltd., National medicine approved: H20080445, 10 mL: 100 mg) was injected with approximately one-quarter of the amount of the capsule fluid. Following another 5 to 10 minutes of washing, 20 to 30 mL of lauromacrogol injection was reintroduced and left in the cystic cavity, with a maximum retention amount of less than 50 mL. The needle was then removed, and the area was bandaged. The vital signs of the patients were closely monitored during and after the operation, and the patients were reexamined before leaving the ultrasound department to ascertain the absence of bleeding and other discomforts. They were then sent back to the clinical department and instructed to rest in bed for speedy recovery.

### 2.4 Follow-up after Lauromacrogol Sclerotherapy

All patients were followed up for over 12 months (mean: 32 ± 11 months). Liver ultrasonography was performed at 1, 3, 6, and 12 months after surgery. The sonographer in charge of sclerotherapy performed the ultrasonography. An ultrasound examination was performed to record the cyst volume, internal structure, and sound transmission after sclerotherapy. The main parameters evaluated included changes in the volume reduction rate (VRR) and symptoms after treatment. The formula employed was as follows: VRR = (initial volume − volume on the follow-up day) / initial volume × 100%. VRR ≥ 50% and <50% at 12-month follow-up were considered effective and ineffective treatments, respectively. To quantify changes in symptoms, patients were followed up at 12 months to fill in the PLD-Q. Clinical symptoms were evaluated according to the patient's improvement rate and a 5-point Likert score, and patients with improvement were defined as effective, while patients with no improvement were classified as ineffective to sclerotherapy. If after the 12-month follow-up, the VRR of the cyst volume after sclerotherapy was <50% or symptoms did not improve, lauromacrogol sclerotherapy or surgery was recommended according to the patient’s preference.

### 2.5 Statistical Analyses

The SPSS 26.0 software was used for statistical analysis. A paired sample *t*-test was used to compare the changes in cyst volume and clinical symptoms before and after lauromacrogol sclerotherapy (at the last follow-up). To analyze the factors related to treatment failure, including age (<55 years old or ≥55 years old), initial maximum cyst diameter (5 cm to 10 cm), initial cyst volume (≤1000 mL) [12], improvement rate, Likert score, and septa formation after cyst sclerosis, the chi-square test was conducted. Multiple logistic regression was then carried out to identify independent factors associated with futility. Statistical significance was set at a *P*-value of <0.05.

## 3 RESULTS

**Fig. (2):**
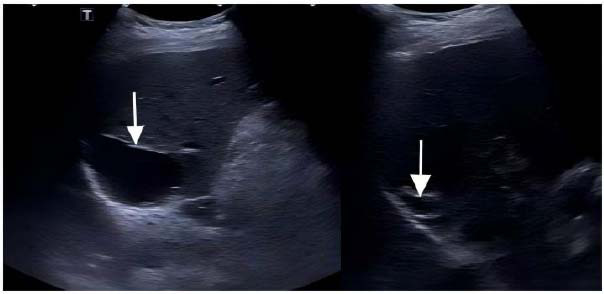
Successful case: Ultrasound image of a 53-year-old man with a hepatic cyst. The initial volume of the liver cyst in the left image was about 330 mL before lauromacrogol hardening treatment. The right image shows that the volume decreased to 9.9 mL (volume reduction rate of 97%) after 12 months of treatment.

The clinical data of 51 patients, including age, sex, and sonographic features of the cysts, are summarized in Table **1**. The changes in cyst volume and improvement rate before and after sclerotherapy were statistically significant (*P* < 0.001). In this study, 96.1% of patients (49/51) with cysts received one sclerotherapy (Fig. **2**), and 3.9% (2/51) received two sclerotherapies (Fig. **1**). Of the two patients who received two treatment courses, one had effective treatment, and another opted for surgical treatment.

The initial volume of the cyst and the volume changes at 1, 3, 6, and 12 months post-treatment are presented in Table **2**. The mean VRR at 1, 3, 6, and 12 months after treatment was 55.3% ± 18.1%, 68.4% ± 17.1%, 77.9% ± 17.1%, and 86.1% ± 15.4%, respectively (Table **2**).

**Fig. (3):**
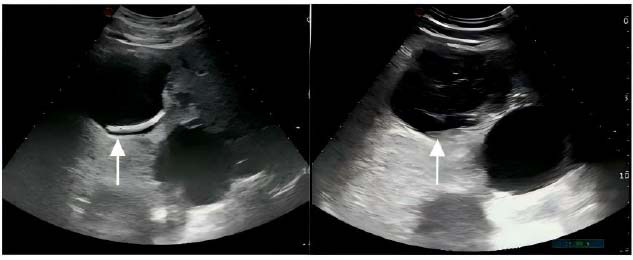
Failed case: Ultrasound image of a 53-year-old man with a hepatic cyst. The initial volume of the hepatic cyst in the left image was approximately 566 mL. The separation in the right image was formed after the ablation, and the volume was approximately 457 mL at 12 months follow-up, with VRR<50%. The patient eventually underwent surgery.

No significant difference was observed in age, sex, and symptom score between patients with VRR ≥50% and patients with VRR <50% at the 12-month follow-up. However, there were significant differences between patients with VRR ≥50% and <50% in the initial cyst volume (p = 0.001), the initial maximum diameter of the cyst (p = 0.005), and septa formation after cyst sclerosis (p = <0.001) (Table **3**). Multivariate logistic regression analysis revealed that septum formation after cyst sclerosis (Fig. **3**) was the only independent factor associated with VRR <50% at the 12-month follow-up (p = <0.001), with an odds ratio of 3.246 (95% confidence interval, 0.784–4.148) (Table **4**).

After lauromacrogol sclerotherapy, five patients developed mild fever, and one had mild pain in the evening. However, these symptoms resolved within 1 week. No serious complications were documented in this study.

## 4 DISCUSSION

The pathogenesis of simple hepatic cysts is attributed to the incomplete development of aberrant bile ducts and lymphatic vessels in the liver during the embryonic period or local lymphatic vessel obstruction secondary to inflammatory epithelial proliferation, resulting in intraluminal secretion retention, with an incidence of 2%–5% [13]. Most patients with hepatic cysts have no clinical symptoms and do not require special treatment as long as they are closely followed. Furthermore, most cysts are often detected during physical examination. Timely and effective treatment is warranted for patients with large cysts and obvious symptoms, such as abdominal pain, abdominal distension, anorexia, and other symptoms [14], which can cause abnormal liver function. In clinical practice, open cyst resection and laparoscopic surgery are generally used for hepatic cyst treatment, but the postoperative recovery of patients undergoing laparoscopic surgery is slow, and the risk for complications is high, which affects patient prognosis.

In recent years, with the development of interventional ultrasound, ultrasound-guided sclerotherapy for hepatic cysts has become the preferred minimally invasive treatment method. Commonly used sclerosants include lauromacrogol and anhydrous alcohol. Although anhydrous alcohol is inexpensive and has a definite therapeutic effect, it can cause severe pain in patients and is associated with a higher incidence of complications. Currently, lauromacrogol is the preferred sclerosant for cyst treatment in clinical practice. Its advantages include simplicity of operation, minimal invasion, cost-effectiveness, and rapid postoperative recovery among patients [15].

Some studies [16-18] have reported that the success rate of lauromacrogol sclerotherapy for hepatic cysts ranges from 80.0% to 96.67%, which is consistent with our study (96.1%; 49/51). Some scholars [17] believe that lauromacrogol sclerotherapy is ineffective in 14%–20% of hepatic cysts. In this study, 3.9% (2/51) of hepatic cysts demonstrated no treatment response (VRR <50% or no improvement in clinical symptoms) at the 12-month follow-up after lauromacrogol sclerotherapy.

In the logistic regression analysis, although the patient’s age, sex, and number of cysts were not correlated with sclerotherapy, the formation of septation after cyst sclerosis was correlated with cyst recurrence. Previous studies have demonstrated that septa formation after cyst sclerosis will affect the results of sclerotherapy [19], leading to ineffective hepatic cyst sclerosis. In this study, the logistic regression analysis revealed that septa formation after cyst sclerosis (*P*<0.001) was an independent risk factor affecting treatment outcomes.

Alicia Furumaya *et al.* [20] reported that the inefficiency of hepatic cyst treatment increased with an increase in the maximum diameter and volume of the cyst. This is attributed to the fact that the larger the hepatic cyst volume, the larger the cyst wall area that needs to be hardened and inactivated, and the amount of lauromacrogol required for lavage increases accordingly. However, the amount of sclerosing agent injected is generally 25%–33.3% of the volume of the cyst fluid extracted, and to prevent the sclerosing agent from leaking into the abdominal cavity, the total amount at one time cannot exceed 50 mL [16, 21]. The folds formed by the cyst wall collapse after cyst fluid extraction were relatively increased. During the flushing of lauromacrogol, a part of the cyst wall could not be completely flushed, resulting in a relative reduction of the cyst wall area in contact with lauromacrogol, thereby increasing the probability of cyst recurrence. However, in this study, the logistic regression analysis did not reveal a correlation between the initial size and maximum diameter of the cyst and cyst recurrence, which could be attributed to the fact that the maximum cyst diameter was <10 cm (the cyst volume was not particularly huge) or the sample size in this study was small.

After lauromacrogol sclerotherapy, ultrasound-guided percutaneous catheter drainage was conducted to extract the accumulated fluid components from the hepatic cysts. Lauromacrogol injection causes aseptic inflammation in the endothelial cells of the inner cyst wall, which causes them to lose their secretory function and promotes thrombosis formation and fibrous tissue proliferation of the cyst capsule, thus playing a role in sclerosis, adhesion, and cyst cavity closure [22, 23]. It is worth mentioning that when lauromacrogol reaches the cyst wall, it induces certain chemical reactions, which may lead to excessive fibrous tissue proliferation, formation of large and small septa affecting the absorption and drainage of exudate cyst fluid, and gradual exudate retention in the cyst cavity, leading to recurrence. In this study, lauromacrogol sclerotherapy resulted in septations in 3.9% of cysts (2/51), which were treated with re-sclerotherapy (1/51) and surgery (1/51). Thus, some scholars believe that [24] postoperative cyst cavity volume is an indicator of whether the cyst wall cells are destroyed and whether the exudate is smoothly discharged after cyst sclerosis.

In this study, one patient with polycystic liver disease was treated with sclerotherapy only for a large cyst. Although the patient’s symptoms were relieved and the cyst did not recur at the end of this follow-up, some studies [25, 26] have reported that the liver of patients with polycystic liver disease is filled with a large number of cysts and the internal structure of the liver is destroyed. Furthermore, in such patients, the cyst may recur after surgery, which might lead to the recurrence of symptoms. Thus, a regular follow-up is required for such patients.

This study has several limitations. First, it was a single-center study. Second, it lacked results from multiple investigations. Third, it had a small sample size. Fourth, no comparison with other treatment regimens was made, and the same regimen was used in all cases. Further studies with more patients and a multicenter design are warranted to verify the efficacy and safety of lauromacrogol sclerotherapy and compare it with liquid sclerotherapy.

## CONCLUSION

Our findings suggest that this minimally invasive technique is effective in reducing cyst volume and alleviating symptoms, with a favorable safety profile. The efficacy of lauromacrogol injection may be influenced by cyst characteristics and patient-specific factors, which warrant further investigation. Future research should focus on optimizing treatment protocols and exploring long-term outcomes to better understand the clinical implications of this intervention.

## Figures and Tables

**Table 1:** Basic information of 51 patients.

**Variable**	**Result**
Age (years), mean ± SD	-
< 55 (n=15)	46 ± 13
≥ 55 (n=36)	64 ± 11
Sex, patient number	-
Male: Female	1.3:1
Follow-up period (months), mean ± SD	32 ± 11
Likert grade, mean ± SD	4.3 ± 0.7
Volume (ml), mean ± SD	-
Initial	539.1 ± 262.3
Last follow-up	101.1 ± 143.6
Initial maximum diameter of a cyst (cm), mean ± SD	8.2 ± 1.3

**Abbreviation:**SD = Standard Deviation.

**Table 2:** Average volume change of 51 cysts during the 12-month follow-up.

Time	Volume (mL) mean ± SD	P	VRR* (%)	VRR < 50% (n, %)	VRR ≥ 50% (n, %)
Initial	539.1 ± 262.2	-	-	-	-
1 month	274.3 ± 197.4	<0.001	55.3 ± 18.1	21 (41.2)	30 (58.8)
3 months	203.8 ± 177.4	<0.001	68.4 ± 17.1	10 (19.6)	41 (80.4)
6 months	148.1 ± 156.5	<0.001	77.9 ± 17.1	6 (11.8)	45 (88.2)
12 months	101.1 ± 143.6	<0.001	86.1 ± 15.4	2 (3.9)	49 (96.1)

**Abbreviation**VRR = Volume Reduction Rate. **Notes:**Data are mean ± standard deviation (percentage). P-value: comparing the current measured volume with the previous measured volume. *The VRR was calculated during each follow-up using the initial volume as the basis. VRR = volume-reduction rate.

**Table 3:** Univariate analysis of factors associated with a volume reduction rate of <50% at 1-year follow-up for 51 cases of hepatic cysts sclerotherapy with lauromacrogol.

Variables	VRR < 50% (n = 6)	VRR ≥ 50% (n = 45)	Total (n = 51)	P (2-Sided)
Age (years)	-	-	-	0.545
≤55	4 (26.7)	11 (73.3)	15	-
>55	2 (5.6)	34 (94.4)	36	-
Sex	-	-	-	0.478
Male	4 (13.8)	25 (86.2)	29	-
Female	2 (9.1)	20 (90.9)	22	-
Likert grade	-	-	-	0.020
>3	3 (6.7)	42 (93.3)	45	-
≤3	3 (50.0)	3 (50.0)	6	-
Initial cyst volume (mL)	-	-	-	<0.001
Initial maximum diameter of a cyst (cm)	-	-	-	<0.001
Cyst underwent sclerosis and became compartmentalized	-	-	-	<0.001

**Note:**Data are presented as number of cysts (percentage). VRR = Volume Reduction Rate.

**Table 4:** Bivariate logistic regression analysis of factors associated with a volume reduction rate of <50% at 1-year follow-up.

**Variables**	**OR**	**95% CI for OR**		**P**
	-	Lower	Upper	-
Sex	-	-	-	-
Female (reference)	1.000	-	-	-
Male	0.200	0.010	3.856	0.286
Age (years)	-	-	-	-
≤ 55 (reference)	1.000	-	-	-
> 55	0.359	0.015	8.409	0.524
Likert grade	-	-	-	-
> 3	0.182	0.027	1.243	0.082
≤ 3 (reference)	1.000	-	-	-
Number of cysts	-	-	-	-
=1	0.150	0.008	2.980	0.213
>1 (reference)	1.000	-	-	-
Cyst underwent sclerosis and became compartmentalized	-	-	-	-
Yes	3.246	0.784	4.148	<0.001
No (reference)	1.000	-	-	-

**Abbreviations:** CI = Confidence Interval; OR = Odds Ratio.

## Data Availability

All data generated or analyzed during this study are included in this published article.

## STANDARDS OF REPORTING

STROBE guidelines were followed.
